# The first true millipede—1306 legs long

**DOI:** 10.1038/s41598-021-02447-0

**Published:** 2021-12-16

**Authors:** Paul E. Marek, Bruno A. Buzatto, William A. Shear, Jackson C. Means, Dennis G. Black, Mark S. Harvey, Juanita Rodriguez

**Affiliations:** 1 Virginia Tech, Blacksburg, USA; 2 Bennelongia Environmental Consultants, Jolimont, Australia; 3 Macquarie University, Sydney, Australia; 4 University of Western Australia, Perth, Australia; 5 Hampden-Sydney College, Hampden Sydney, USA; 6 La Trobe University, Melbourne, Australia; 7 Western Australian Museum, Perth, Australia; 8 Australian National Insect Collection, Canberra, Australia

**Keywords:** Taxonomy, Entomology

## Abstract

The name “millipede” translates to a thousand feet (from *mille* “thousand” and *pes* “foot”). However, no millipede has ever been described with more than 750 legs. We discovered a new record-setting species of millipede with 1,306 legs, *Eumillipes persephone*, from Western Australia. This diminutive animal (0.95 mm wide, 95.7 mm long) has 330 segments, a cone-shaped head with enormous antennae, and a beak for feeding. A distant relative of the previous record holder, *Illacme plenipes* from California, it belongs to a different order, the Polyzoniida. Discovered 60 m below ground in a drill hole created for mineral exploration, *E. persephone* possesses troglomorphic features; it lacks eyes and pigmentation, and it has a greatly elongated body—features that stand in stark contrast to its closest surface-dwelling relatives in Australia and all other members of its order. Using phylogenomics, we found that super-elongation (> 180 segments) evolved repeatedly in the millipede class Diplopoda. The striking morphological similarity between *E. persephone* and *I. plenipes* is a result of convergent evolution, probably for locomotion in similar soil habitats. Discovered in the resource-rich Goldfields-Esperance region and threatened by encroaching surface mining, documentation of this species and conservation of its habitat are of critical importance.

## Introduction

Among the earliest animals to breathe atmospheric oxygen^1^ and with some extinct species that grew to two meters in length^2^, millipedes have lived on this planet for more than 400 million years. Important as decomposers in terrestrial ecosystems^3^, primary knowledge of millipede diversity lags tremendously behind other animal groups. With fossil taxa dated to the Cretaceous^4,5^, the millipede order Polyzoniida includes ca. 70 species with a distribution on all continents except Antarctica. Some of its members exhibit parental care of eggs^6^, others ooze chemical defenses containing alkaloids^7^ that are sequestered by poison frogs^8,9^, and some species roll into a ball for protection^10^. Hatchlings emerge from the egg with four pairs of legs, and continuously add segments during development for an indeterminate period of time, even after adulthood^11^. Polyzoniidans have never before been observed in deep soil habitats, and most occur in surficial microhabitats of decaying wood and other detritus. Previously known Australian polyzoniidans have at most 400 legs, eyes with 1–3 ommatidia, most with at least some dark pigmentation, a flat wide body (relative to others in the order), and occurrence in epigean microhabitats^12^. However, distantly related millipedes, such as *Illacme* species and other members of the family Siphonorhinidae (order Siphonophorida), have been recorded from as deep as 11.5 m below the soil surface^13,14^. These species possess a suite of adaptations, such as a thin elongated body with up to 750 legs, no eyes, massive antennae, and lack of pigmentation. No siphonorhinid species have been recorded from Australia, and the geographically closest siphonorhinids occur in Madagascar and Indonesia^13,14^.

Here we report the discovery of *E. persephone*, the first super-elongated millipede known from Australia, and the new world record holder of the animal with greatest number of legs. It belongs to the family Siphonotidae (order Polyzoniida), yet appears similar to super-elongated millipedes in the order Siphonophorida. *Eumillipes persephone* lives deep underground, and it was only discovered by surveying geological drill holes originally created for mineral exploration that provided access to a cryptic and previously unexplored underground habitat.

## Results

**Figure 1:**
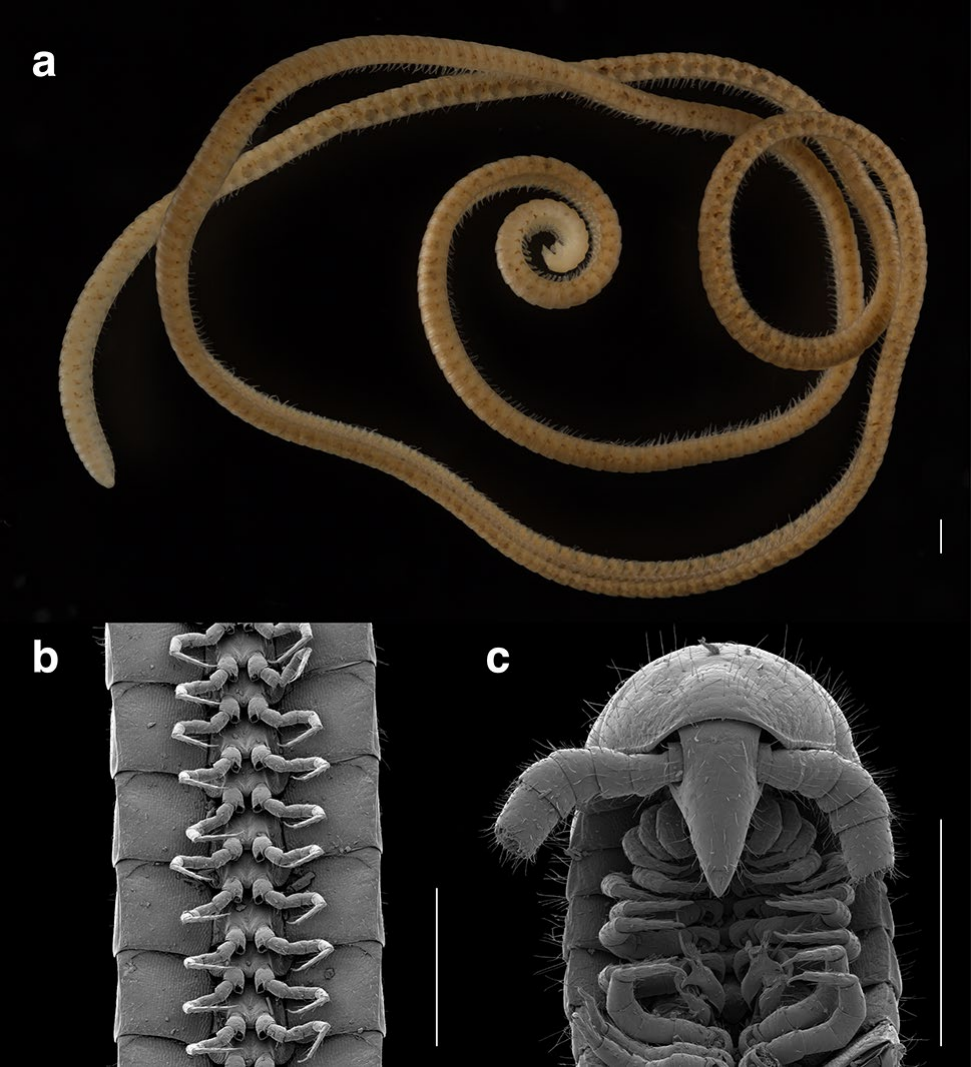
The leggiest animal on the planet, *Eumillipes persephone*, from Australia. (**A**) female with 330 segments and 1,306 legs (paratype specimen, T147124). (**B**) ventral view of legs (male holotype, T147101). (**C**) dorsal view of head and ventral view of gonopods (male holotype, T147101). Scale bars, 0.5 mm.

In August 2020, in the Goldfields region of Western Australia, we discovered a pale, thread-like millipede with 1,306 legs at a depth of 60 m in a drill hole created for mineral exploration (Fig. 1). In total, 56 4–81 m deep 150 mm-diameter drill holes located at a site 100 km WSW of Norseman, Western Australia (within the Great Western Woodlands) were sampled for millipedes and other subterranean fauna, according to techniques described in ref.^16^. These geological drill holes provided a portal to access an interstitial habitat composed of banded iron formations and mafic volcanic rocks, which are known to harbor troglophilic fauna^16,17^. Eight individuals of the new species were collected from troglofauna traps at depths between 15–60 m from three drill holes (450 m apart), including five individuals from a trap set at a depth of 60 m. Two juveniles were collected in April 2020 and another one in January 2021, always from troglofauna traps, attesting to the species’ true deep subterranean life.

**Figure 2:**
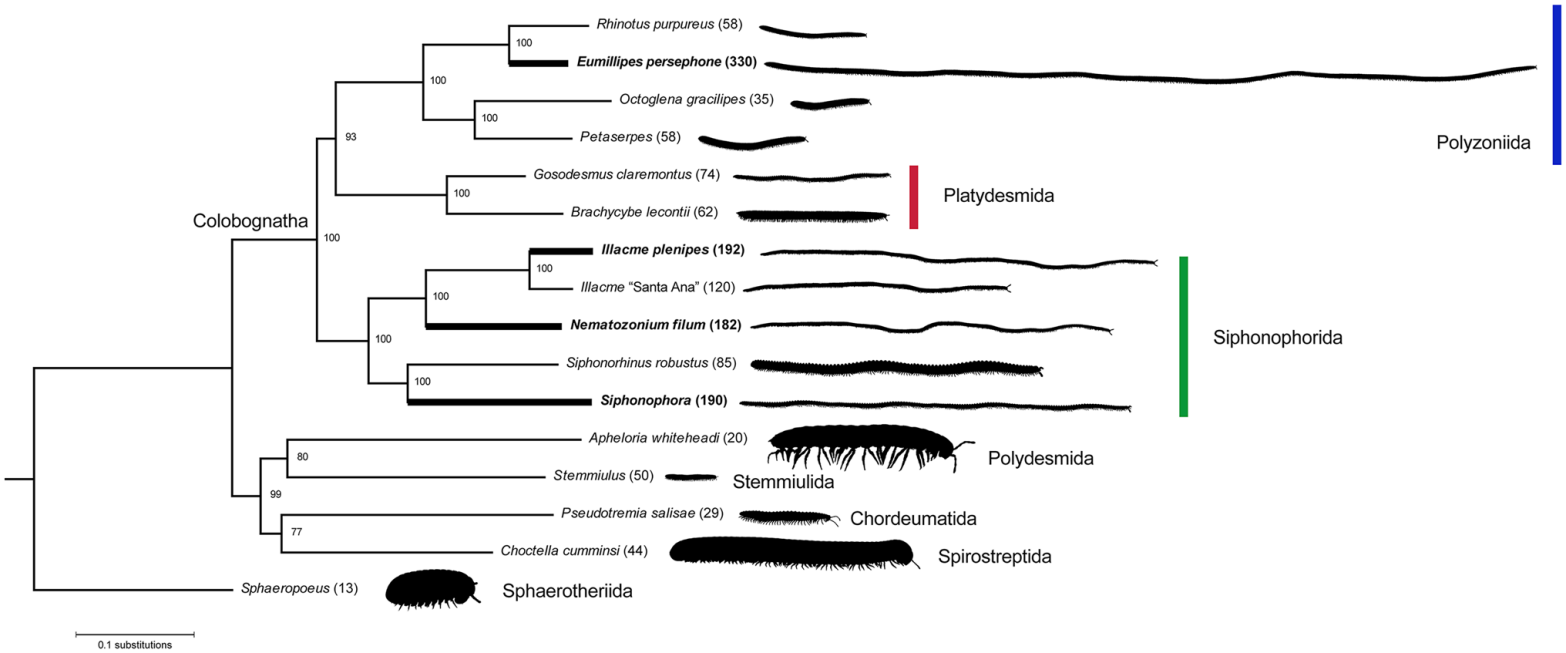
Phylogenomic estimation of the evolutionary history of colobognath millipedes. Super-elongation (> 180 segments) is well known from the order Siphonophorida, including *Illacme plenipes* with 192 segments and 750 legs, but it has independently evolved in the Australian order Polyzoniida with *Eumillipes persephone* bearing up to 1,306 legs and 330 segments. This feature (dark branches) has evolved between two and four times in diplopods based on a character state reconstruction using parsimony. Maximum likelihood phylogeny of 312 orthologous sequences with Polydesmida, Stemmiulida, Chordeumatida, Spirostreptida, and Sphaerotheriida as outgroup taxa. Julida, Spirobolida, and other diplopod orders omitted from the analysis. Support values on nodes are bootstrap supports. Numbers in parentheses after species names are the maximum segment count for the taxon. Species with super-elongation present denoted in bold. Millipede silhouettes sized relative to one other. Diagram created with Adobe Illustrator 2021 (adobe.com/products/illustrator.html).

The specimens superficially appeared to be members of the Siphonorhinidae, due to phenotypic similarity to *Illacme plenipes* Cook & Loomis, 1928. We used scanning electron microscopy and genome sequencing to conclude that the remarkable anatomical similarity between *E. persephone* and *I. plenipes* is a result of convergent evolution of super-elongation (> 180 segments), which we here reveal that has originated at least twice in the millipede class Diplopoda (Fig. 2). *Eumillipes persephone* appears unlike any other species in the order Polyzoniida. Its highly elongated and narrowed body, lack of eyes, massive antennae, shortened legs, and lack of pigmentation comprise a suite of characters consistent with a fossorial *bauplan* that has repeatedly evolved in the Diplopoda^18,19^.

### Taxonomy section

Class **Diplopoda** de Blainville in Gervais, 1844

Subterclass **Colobognatha** Brandt, 1834

Order **Polyzoniida** Cook, 1895

Family **Siphonotidae** Cook, 1895

Tribe **Rhinotini** Hoffman, 1977

### *Eumillipes*, Marek new genus

http://zoobank.org/NomenclaturalActs/a258f64e-0d4c-4941-be20-0b5d78e1078e.

### Type species

*Eumillipes persephone* Marek, new species.

### Generic placement and diagnosis

The genus *Eumillipes* is placed in the order Polyzoniida, family Siphonotidae, tribe Rhinotini based on the following characters^10,20^; whether the character is diagnostic for the order (O), family (F), or tribe (T) is denoted in parentheses. Head capsule small, conical, elongated into a sharp snout (O) (Fig. 1C, Supplementary Fig. S1A–C). Prozonites and metazonites of trunk rings the same width (O) (Supplementary Fig. S2A); prozonites not narrow as in the Siphonophorida. Rings smooth (Supplementary Fig. S2B), covered with neither cuticular ornaments nor long setae as in the Siphonophorida. Vertex of head with two macrosetae (O) (Supplementary Fig. S1C). Antennae thick with equally sized antennomeres (O) (Fig. 1C, Supplementary Fig. S3A); antennae not strongly elbowed between antennomeres 3, 4 as in the Siphonorhinidae (Siphonophorida). Gnathochilarium reduced to three sclerites: mentum; left, right stipes (O). Ozopores located far in from lateral edges of tergites; placed two-thirds the distance from midline laterally to tergal margins (F) (Supplementary Fig. S2D). Vasa deferentia open through small conical lobes (penes) on the posterior surfaces of the second leg coxae (F). Telson a complete ring around the anal valves (F) (Supplementary Fig. S2D). Tarsal claw with long sigmoid-shaped accessory claw at its base that exceeds the claw in length (F) (Supplementary Fig. S1D). The genus differs from other siphonotid genera by the following characters. Anterior gonopods (i.e., 9th leg pair) strongly modified with podomeres 3–4 fused, not leg-like as in Siphonotini genera (T) (Fig. 1C, Supplementary figs. S4, S5A). Apex of anterior gonopods distinctly bifurcated into two processes (Fig. 1C, Supplementary figs. S4, S5A), not a single process as in the genera *Rhinotus* Cook, 1896 and *Siphonoconus* Attems, 1930.

### *Eumillipes persephone* Marek, new species

http://zoobank.org/NomenclaturalActs/0AFB7037-E517-4D05-804B-D9AE1C7B3F47

### Diagnosis

Adults of *Eumillipes persephone* can be differentiated from other polyzoniidan genera and species (and commonly encountered millipedes co-occurring with *E. persephone* in Western Australia) based on the combination of the following characters. Body extremely long and thread-like (width: ♂ 0.92 mm; ♀ 0.95 mm; length: ♂ 54.7 mm; ♀ 95.7 mm). Exoskeleton uniformly pale, cream-colored (Fig. 1A, Supplementary figs. S6–S8)—with neither dark pigmentation nor longitudinal, nor transverse stripes as in surface-dwelling species (Supplementary Fig. S9). Adult millipedes with an exceptional number of rings and legs: ♀ up to 330 rings and 1,306 legs, and ♂ up to 208 rings and 818 legs (Supplementary tab. S1). Head cone-shaped and eyeless (Fig. 1C, Supplementary Fig. S1A–C)—lacking eyes as are present in surface-dwelling species. Ninth and 10th leg pairs modified into gonopods (Fig. 1C, Supplementary figs. S4, S5). Anterior gonopods (9th leg pair) highly modified, not leg like, and distinctly bifurcated into two processes (Fig. 1C, Supplementary figs. S4, S5A). Medial process of the anterior gonopods saddle-shaped—not pointed and recurved as in *Siphonotus flavomarginatus* Attems, 1911^21^. Lateral process sheath-like and cupping the medial process. Posterior gonopods (10th leg pair) stylet-like, and in repose threaded through the bifurcated anterior gonopods (Fig. 1C, Supplementary figs. S4, S5B). Sterna of gonopods with four long, slender, curved setae apically studded with spinules (Supplementary figs. S4B, S5A).

#### Material examined

Male holotype (WAM T147101), two female and one male paratypes (WAM T147100, T147124, T146684), and two juveniles (WAM T147122, T147123) from Western Australia, ca. 100 km WSW of Norseman, 32° 32′ 05.9" S, 120° 47′ 42.74" E, 27 May–4 August 2020, collected by A.J. Mittra and L.P. Masarei (Western Australian Museum).

#### Variation

Males have fewer segments and legs; specimen T147101 has 198 segments and 778 legs (Supplementary Fig. S7), and T147100 has 208 segments and 818 legs (Supplementary Fig. S8). Female specimen T147124 possesses 330 segments and 1,306 legs (Fig. 1A, Supplementary Fig. S6), and T146684 has 253 segments and 998 legs.

#### Etymology

The genus is named because it is the first true millipede with more than 1000 legs. The name *Eumillipes* is a combination of the Greek *eu-*, meaning ‘true’; Latin *mille*, ‘thousand’; and Latin *pes*, ‘foot’. It is to be treated as a noun. The species epithet is derived from the Greek mythological goddess of the underworld, Persephone, who was originally from the surface but was taken to the underworld by Hades.

## Discussion

From studies by Manton^22^ and video footage of siphonorhinid millipedes, the locomotive biomechanics of super-elongated millipedes has only been studied rudimentarily. Navigating by its enlarged antennae (Fig. 1C, Supplementary Fig. S3A, B) equipped with up to five sensillum types^15^, the millipede’s elongated trunk with telescopic segments facilitates burrowing in a three-dimensional soil matrix. Its antennae wave independently to locate an opening; once a suitable gap is identified, the millipede enters and walks through. The flexible segments are compressible to squeeze through narrow fissures. The continuous metachronal gait and musculoskeletal action of the segments, which are composed of concentric tubular rings that slide within one another, provide continuous pushing force; in combination with the legs, longitudinal (Supplementary Fig. S10C) and oblique muscles pull the rings together and provide forward locomotion^22^. This way of burrowing resembles that used by geophilomorph centipedes (another group of greatly elongated myriapods that includes the leggiest centipede, *Gonibregmatus plurimipes* Chamberlin, 1920 from Fiji with 191 rings and 382 legs) and earthworms. In contrast with millipedes with incompressible rings such as “bulldozer” ecomorphs that walk in a single plane propelled by the additive force of their legs^22^, colobognath millipedes are able to wind their way through a three-dimensional matrix and can simultaneously walk in up to eight different planes. This telescoping locomotion, by sliding trunk segments (Supplementary Fig. S2A) coupled with the thrust of the legs, propels the animal through a varied and unpredictable underground microhabitat^22^, and the increase in leg number likely contributes more pushing power to force through small crevices and openings. Super-elongation in the Diplopoda has repeatedly evolved in taxa that live in these microhabitats, such as in Siphonorhinidae (*I. plenipes* with 192 segments and *Nematozonium filum* Verhoeff, 1939 with 182 segments), Siphonophoridae (*Siphonophora millepeda* Loomis, 1934 and *Siphonacme lyttoni* Cook & Loomis, 1928 each with 190 segments), and *E. persephone*. Each additional ring provides extra locomotory thrust^23^, and *E. persephone*’s up to 330 rings may be specially adapted to locomotion in its comparatively deep soil microhabitat at 60 m—five-fold greater than the maximum depth of *Illacme* species. Trunk super-elongation may also serve to lengthen the digestive canal to increase the absorptive surface area and assimilation efficiency in a resource-limited subterranean habitat, as may be the case for *I. plenipes*, which additionally has an even longer corkscrew-shaped gut than the trunk itself^15^. The diet of *E. persephone* is unknown, but may be similar to other primarily fungivorous colobognath millipedes, such as the platydesmidan *Brachycybe lecontii* Wood, 1864 that feeds on up to 176 different genera of fungi^24^.

Current alpha-level knowledge of Australian Siphonotidae is fragmentary. Although an unpublished dissertation from 1994 documented eight genera and 42 species of the family^25^, taxonomy of the group remains antiquated with two genera and five species currently listed from the continent described about a century ago^21,25,26^. Although the phenotypic divergence of *E. persephone* from surface-dwelling family relatives appears to be associated with adaptations to life underground, extraordinary similarity of its gonopods (species-diagnostic features in diplopods) with *Siphonotus flavomarginatus* from Torbay, Western Australia, indicates a genus-level affiliation. Close similarity of gonopod anatomy with extremely divergent somatic features suggests rapid evolution of troglomorphic traits.

Invertebrates that live below the Earth’s surface comprise a cryptic and diverse fauna. Although they are challenging to observe and document, notable recent discoveries include beetles and millipedes in Brazilian iron ore caves^27,28^, snake millipedes of mesovoid shallow substratum of Spain^29^, and spiders from caves of the Edwards Plateau of Texas^30^. These habitats are repositories of incredibly rich, but obscure biodiversity. These underground habitats, and their inhabitants, are critically understudied, despite their ecological importance in filtration of groundwater and screening of environmental toxins. The subterranean conditions of Western Australia may consistently be at a much lower temperature and higher humidity and house an evolutionarily closely related mesic-adapted troglofauna. Despite surface temperatures in excess of 46 °C and less than 300 mm/year of precipitation^31^, the groundwater in the sites where *E. persephone* was collected never exceeded 22 °C. Surface climatic conditions fluctuated considerably across hundreds of millennia, but underground conditions probably remained comparatively stable. Troglophilic species often represent ancient vestiges of formerly widespread lineages that now persist in subterranean refugia^32^. Although the date of the split between the clade containing *E. persephone* and the one containing *Siphonotus flavomarginatus* is uncertain, divergence may be linked to aridification of the continent between 15–1.75 Ma. The so-called “climatic relict” theory has been useful to explain other such close relationships between troglophilic and epigean taxa, such as springtails, isopods, schizomids, pseudoscorpions, and ground beetles in Western Australia^17,33^. This theory suggests that cool-adapted epigean ancestors were gradually driven underground where conditions remained favorable, whilst the surface dried and became uninhabitable; following aridification, epigean lineages underwent extinction thereby fostering allopatric speciation^33^. A similar theory has been invoked for faunal radiations in caves, where analogous extreme adaptive shifts also occur accompanied by morphological change: eyelessness, elaboration of mechanical sensation, lack of pigmentation, and leg elongation. Elongated limbs in troglobionts have been hypothesized to have originated for more efficiently traversing expansive cavernous space for scarce food and mates^34^. In the case of *E. persephone*, and its micro-cavernous habitat, the remarkable number of legs may be associated with a comparable search for limited resources in its “micro-cave”-like interstitial habitat. Short legs are likely advantageous in the small cavities of such a habitat, and perhaps compensated for by an increase in their number, which would maintain pushing power.

Located in the Yilgarn Craton, the Eastern Goldfields Province of Western Australia composes a surficial habitat of shrublands (Supplementary Fig. S11) overlaying an ancient terrane that is among the oldest emergent land masses (2.6–4.4 billion years, ref.^35^). A large quantity of Australia’s gold, nickel, and other minerals are extracted from the Goldfields, and intensive surface mining has occurred in the area for more than a century. Mineral exploration includes surveying underground via millions of drill holes that produced cores for elemental analysis in the region. These holes, which are later rehabilitated, provide fleeting access to sample an expansive vadose zone which houses a subterranean biodiversity hotspot^17,36^.

Whether troglomorphic species of *Eumillipes* exist in other parts of Western Australia is an uncertain, but potentially rich avenue of discovery. The unexpected discovery of *E. persephone* and other troglomorphic animals from the Great Western Woodlands^37,38^, within the Eastern Goldfields Province of Western Australia, highlights the region as an exceptional repository of biodiversity.

## Methods

### Collections

Millipedes were collected from drill holes according to ref.^16^. Drill holes were preexisting and bored by mining companies for mineral exploration. 56 4–81 m deep 150 mm diameter wide drill holes were sampled with specifically designed traps (trogtraps) that are lowered into holes to sample subterranean invertebrates. Trogtraps consist of cylindrical PVC traps with numerous apertures, which are baited with moist leaf litter and lowered on nylon cord to varying depths. The leaf litter bait is wetted, allowed to decompose over weeks or months and sterilized via microwaving. Holes in the trogtraps are capped at the surface while traps are set to minimize the collection of surface invertebrates. Trogtraps were left in the field for approximately two (but up to five) months for troglofauna to be attracted to the aroma of decaying vegetation. At the end of the sampling period, traps were carefully pulled out of each hole and the contents placed in zip lock bags, allowing enough oxygen for transit back to the laboratory. In the laboratory, troglofauna were extracted from the leaf litter in traps using Tullgren funnels under incandescent lamps: light and heat from the lamps drives the animals out of the litter towards the base of the funnel and into a collection vial containing 100% ethanol. Samples were obtained under the auspices of scientific research permit number BA27000150 from the Department of Biodiversity, Conservation and Attractions of Western Australia. Millipede specimens were fixed and preserved in ethanol and deposited in the Western Australian Museum, Perth, Australia with the following catalog numbers T147100, T147101, T147122–T147124, and T146684.

### DNA extraction and sequencing

Tissues were dissected from ethanol preserved specimens and DNA extracted from them according to ref.^39^. 6–10 rings were removed and pulverized with a micro-pestle in 200 uL lysis buffer containing proteinase K using a Qiagen DNeasy kit. Purified genomic DNA was quantified and quality assessed using a Nanodrop spectrophotometer and Agilent 5400 fragment analyzer. Genomic DNA was randomly fragmented by sonication, Illumina adapters ligated, amplified by PCR, and libraries constructed followed by quantification and a quality-control step. The libraries for each sample were pooled to 1–3 sequencing lanes to obtain a targeted 9 GB of data using an Illumina Novaseq 6000 two channel platform with paired-end 150 bp reads. Standard DNA cytochrome oxidase I gene (COI) barcodes for the holotype specimen, and two juveniles were sequenced with the primer pair LCO1490 and HCO2198 according to methods in ref.^39^ (NCBI accession #: OK602741).

### Phylogenomics

Raw reads of genomes were data filtered to remove adapter sequences, reads containing > 10% N, and low-quality reads with > 50% of the read with phred ≤ 5. FastQC Version 0.11.9^40^ was used to evaluate quality scores, GC content, sequence length distribution, and duplicated and overrepresented sequences. Reads were deposited in the National Center for Biotechnology Information SRA with the BioProject accession number, PRJNA769673, and the following catalog numbers: *Sphaeropoeus* Brandt, 1833 (MPE02963), *Choctella cumminsi* Chamberlin, 1918 (MPE01425), *Pseudotremia salisae* Lewis, 2000 (MPE04962), *Stemmiulus* Gervais, 1844 (MPE04756), *Apheloria whiteheadi* (Shelley, 1986) (MPE05046), *Siphonophora* Brandt, 1837 (MPE04720), *Siphonorhinus robustus* (Attems, 1938) (MPE05063), *Nematozonium filum* Verhoeff, 1939 (T129482), *Illacme* “Santa Ana” (MPE04624), *Illacme plenipes* Cook & Loomis, 1928 (SPC001187), *Brachycybe lecontii* Wood, 1864 (MPE03842), *Gosodesmus claremontus* Chamberlin, 1922 (SPC00941), *Petaserpes* Cope, 1870 (MPE00887), *Octoglena gracilipes* (Loomis, 1971) (Sass.A.230), *Eumillipes persephone* (T147101), *Rhinotus purpureus* (Pocock, 1894) (MPE05072). Trimmomatic Version 0.39^41^ was used to trim low quality bases from the end of the reads using ‘trailing 28’, and omit short reads using ‘minlength 75’. aTRAM version 2.0^42^ was used to assemble sequencing reads using the python script ‘atram_preprocessor.py’ onto a set of 312 orthologous genes (matrix 4 from ref.^4^). A set of BLAST libraries containing sequence shards were assembled with ‘atram.py’ implementing the assembler Velvet Version 1.2.10^43^. Assembled genes were translated and placed in individual matrices with exonerate Version 2.2.0^44^ in the aTRAM module stitcher, ‘atram_stitcher.py’. Amino acids were independently aligned with Mafft V7.407^45^, allowing for automatic selection of appropriate strategy based on data size, using the Smith-Waterman algorithm for alignment and performing 1000 cycles of iterative refinement. Alignments were then visually inspected, manually refined and concatenated using Geneious Prime 2020.2. A Maximum Likelihood approach was undertaken for phylogenetic reconstruction using IQ-TREE V1.7.0b7^46^ with a partitioned scheme, testing the model of molecular evolution for each partition and an ultrafast bootstrap analysis of 1,000 replicates^47^.

### Morphology

The holotype male (T147101) and paratype female (T146684) specimens were prepared for scanning electron microscopy (SEM) by removing the anterior, middle, and posterior 10 rings, and air drying them at room temperature until the ethanol preservative evaporated. Dissected structures were then placed on double-sided carbon tape on a 12.7 mm aluminum SEM stub, and sputter coated with a 40-nm layer of palladium and platinum in a Leica EM ACE600 plasma sputter coater. Micrographs were taken with a FEI Quanta scanning electron microscope at the Nanoscale Characterization and Fabrication Laboratory in the Institute for Critical Technology and Applied Science at Virginia Tech. Examination of general morphology for descriptive taxonomy was carried out on a Leica M125 stereomicroscope (10–125X) and a Leica DM500 brightfield microscope (50–400X).

## Supplementary Information

Supplementary Information.

## Data Availability

The datasets analyzed in the current study are available from Virginia Tech's Digital Repository (10.7294/16850347) and the National Center for Biotechnology Information (https://www.ncbi.nlm.nih.gov/sra/PRJNA769673). This publication and its nomenclatural acts have been registered in Zoobank (www.zoobank.org), the official registry of the International Commission on Zoological Nomenclature. The LSID (Life Science Identifier) of the article is http://zoobank.org/References/6829E779-7AE8-476E-99FF-DD2311C70199.
