# Supplementary material for: The first true millipede—1306 legs long
Source: Sci Rep. 2021 Dec 16;11:23126. doi: 10.1038/s41598-021-02447-0 (PMC8677783; doi:10.1038/s41598-021-02447-0)
Supplement: Supplementary file 1 — Supplementary Information. [file 41598_2021_2447_MOESM1_ESM.docx]

**Supplementary Information for**

**The first true millipede—1,306 legs long**

Paul E. Marek*, Bruno Buzatto, William A. Shear, Jackson C. Means, Dennis G. Black, Mark S. Harvey, Juanita Rodriguez

*Corresponding author: Paul E. Marek

Email: paulemarek@gmail.com

**Supplementary Text**

**Phylogenetic results.** The ultrafast bootstrap consensus tree largely resembled the topology obtained by ref. *1*, with Pentazonia branching-off early and sister to Helminthomorpha. Within Helminthomorpha, Colobognatha is sister to Eugnatha. Within Eugnatha, Juliformia is sister to Stemmiulida+Polydesmida. Within Colobognatha, Siphonophorida is sister to Polyzoniida+Platydesmida. *Eumillipes persephone* is confidently placed within Polyzoniida and closely related to *Rhinotus* (Figure 2)*.* The evolution of super-elongation (>180 segments) was reconstructed on the phylogeny with parsimony character optimization criterion in Mesquite [*2*].


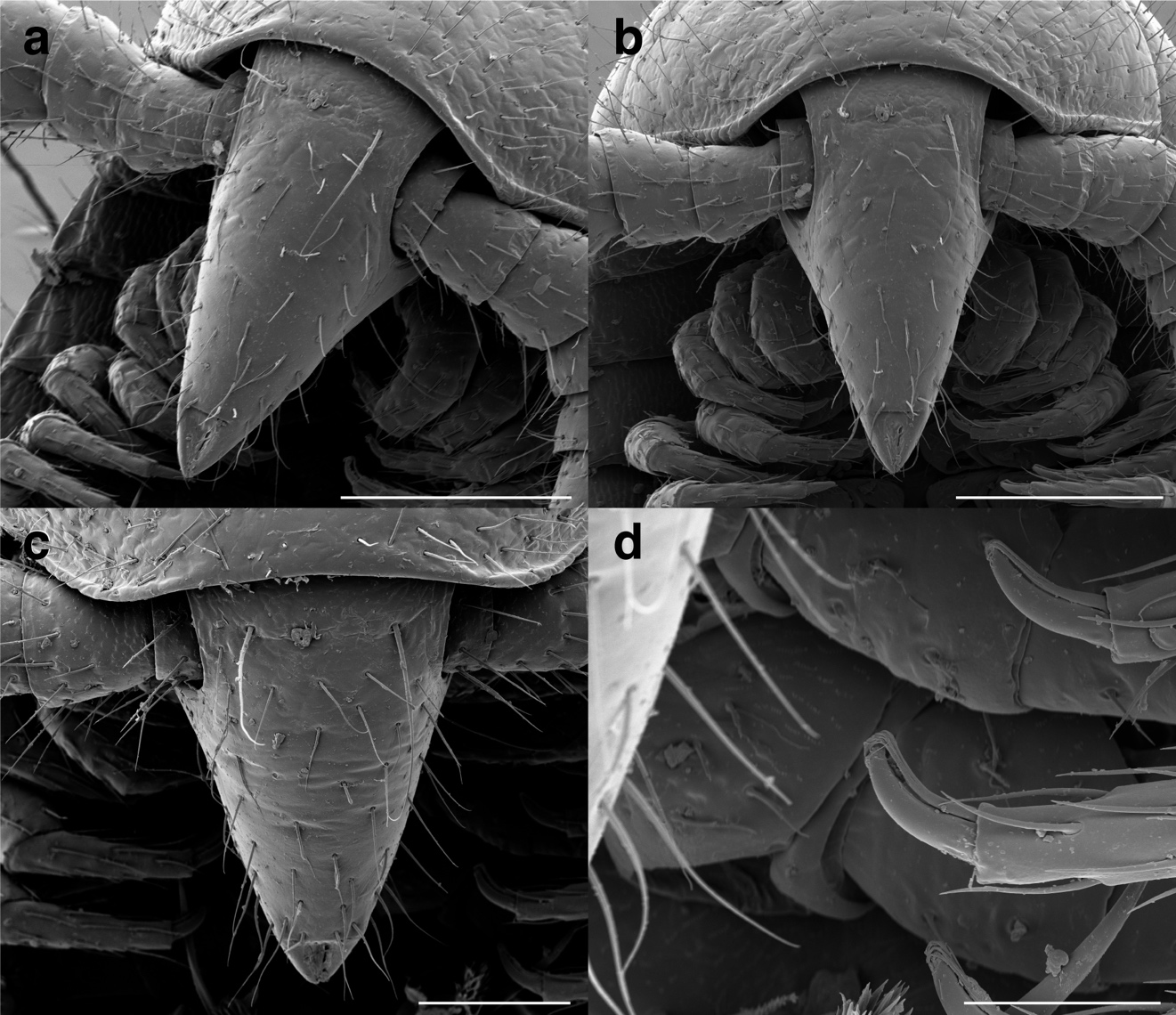


**Supplementary figure S1.** *Eumillipes persephone* male holotype (T147101). A. Head, oblique view, showing eyeless cone-shaped head. (Scale bar = 200 μm). B. Head, dorsal view. (Scale bar = 200 μm). C. Head, dorsal view, showing two macrosetae. (Scale bar = 100 μm). D. Seventh leg with tarsal claw with long sigmoid-shaped accessory claw at its base that exceeds the claw in length. (Scale bar = 50 μm).


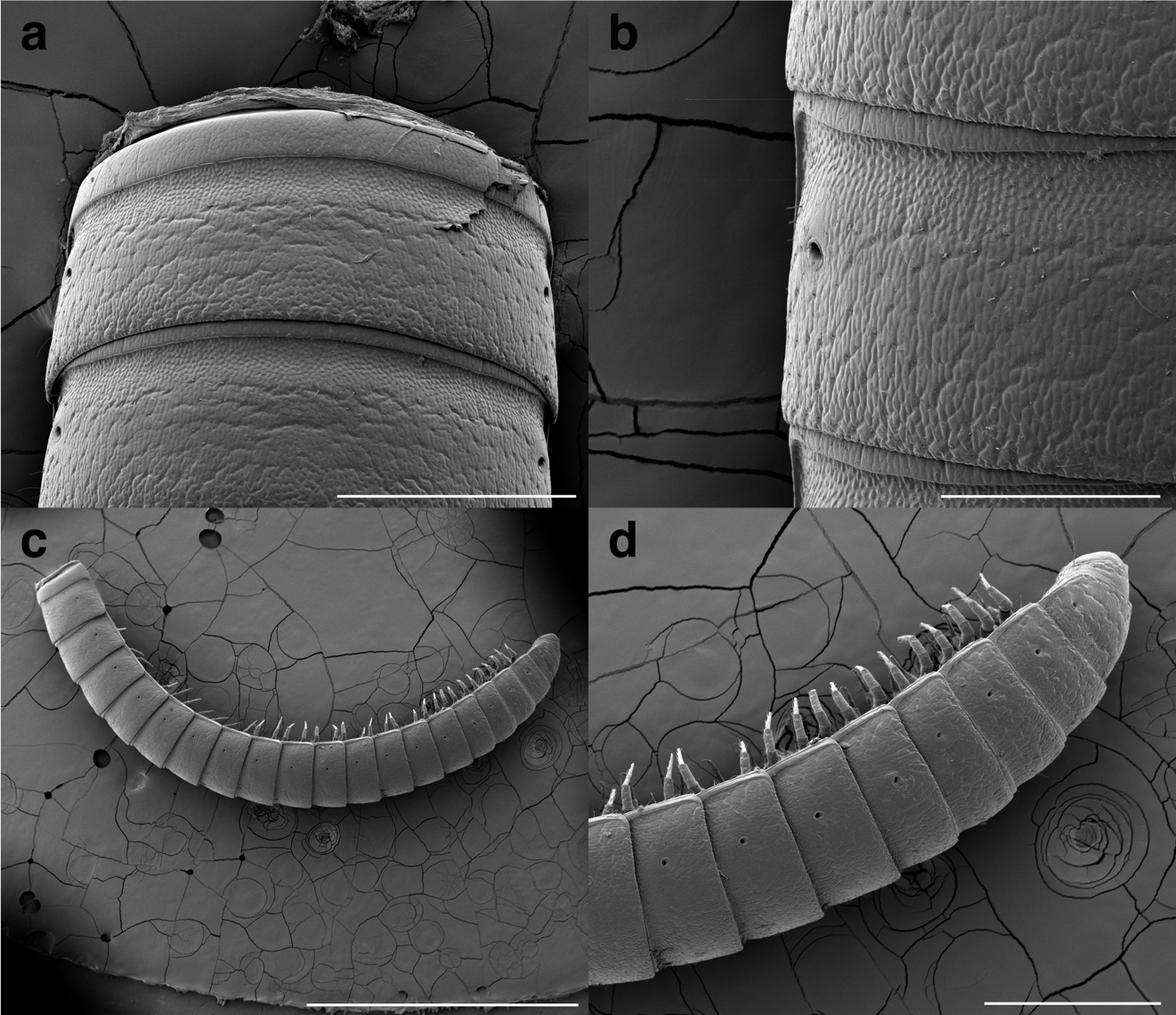


**Supplementary figure S2.** *Eumillipes persephone* male holotype (T147101). A. Mid-length trunk segments, dorsal view, with prozonites and metazonites of trunk rings the same width. (Scale bar = 400 μm). B. Mid-length trunk segment, dorsal view of ozopore. (Scale bar = 200 μm). C. Posterior-most 19 segments + telson, lateral view (Scale bar = 2 mm). D. Posterior-most 9 segments + telson, lateral view. (Scale bar = 500 μm).


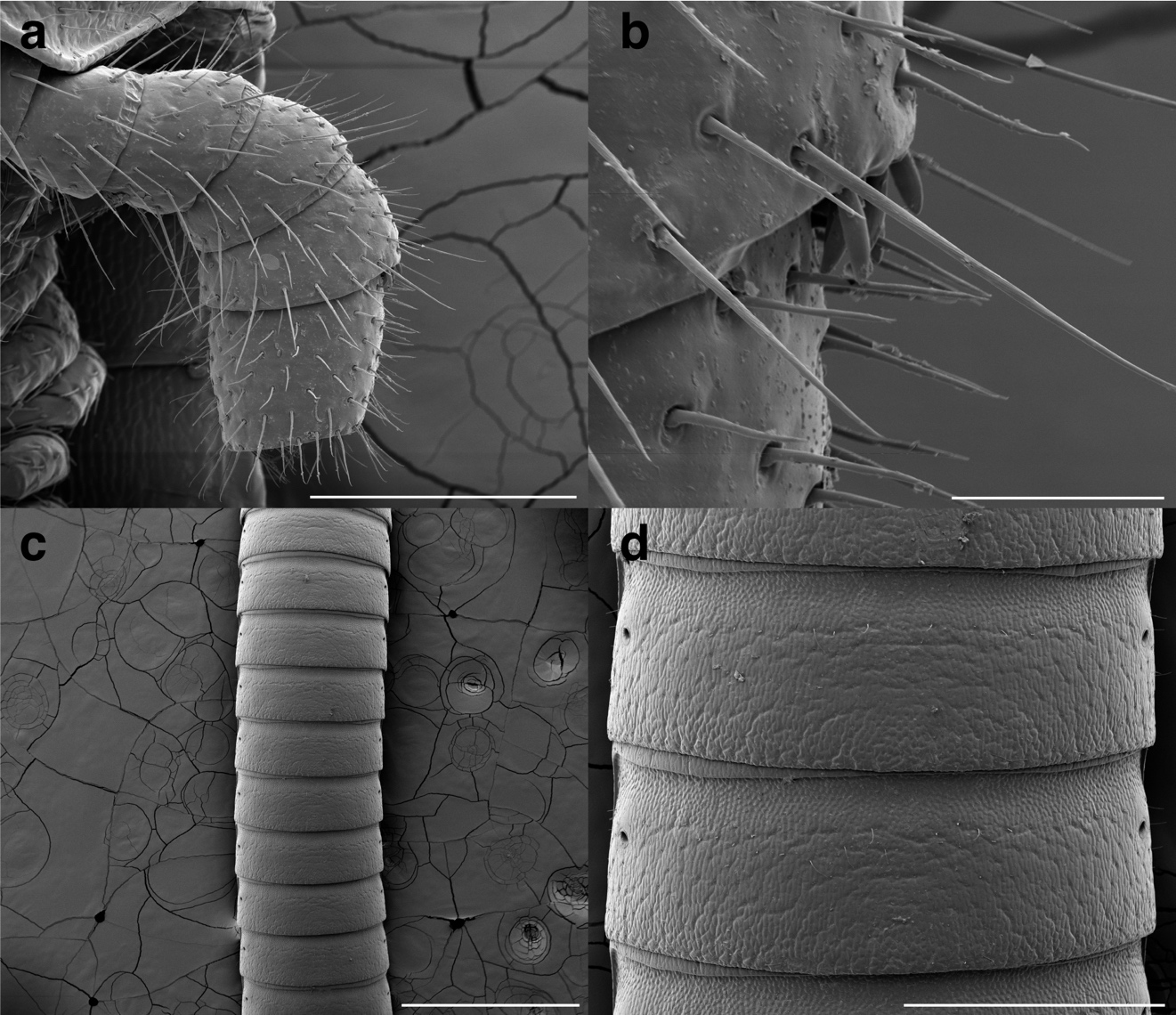


**Supplementary figure S3.** *Eumillipes persephone*. A. Female paratype (T146684), left antenna. (Scale bar = 200 μm). B. Female paratype (T146684), left antenna showing small basiconic sensilla on antennomere 6. (Scale bar = 30 μm). C. Male holotype (T147101), mid-length trunk segments, dorsal view. (Scale bar = 1 mm). D. Male holotype (T147101), mid-length trunk segments, dorsal view. (Scale bar = 400 μm).


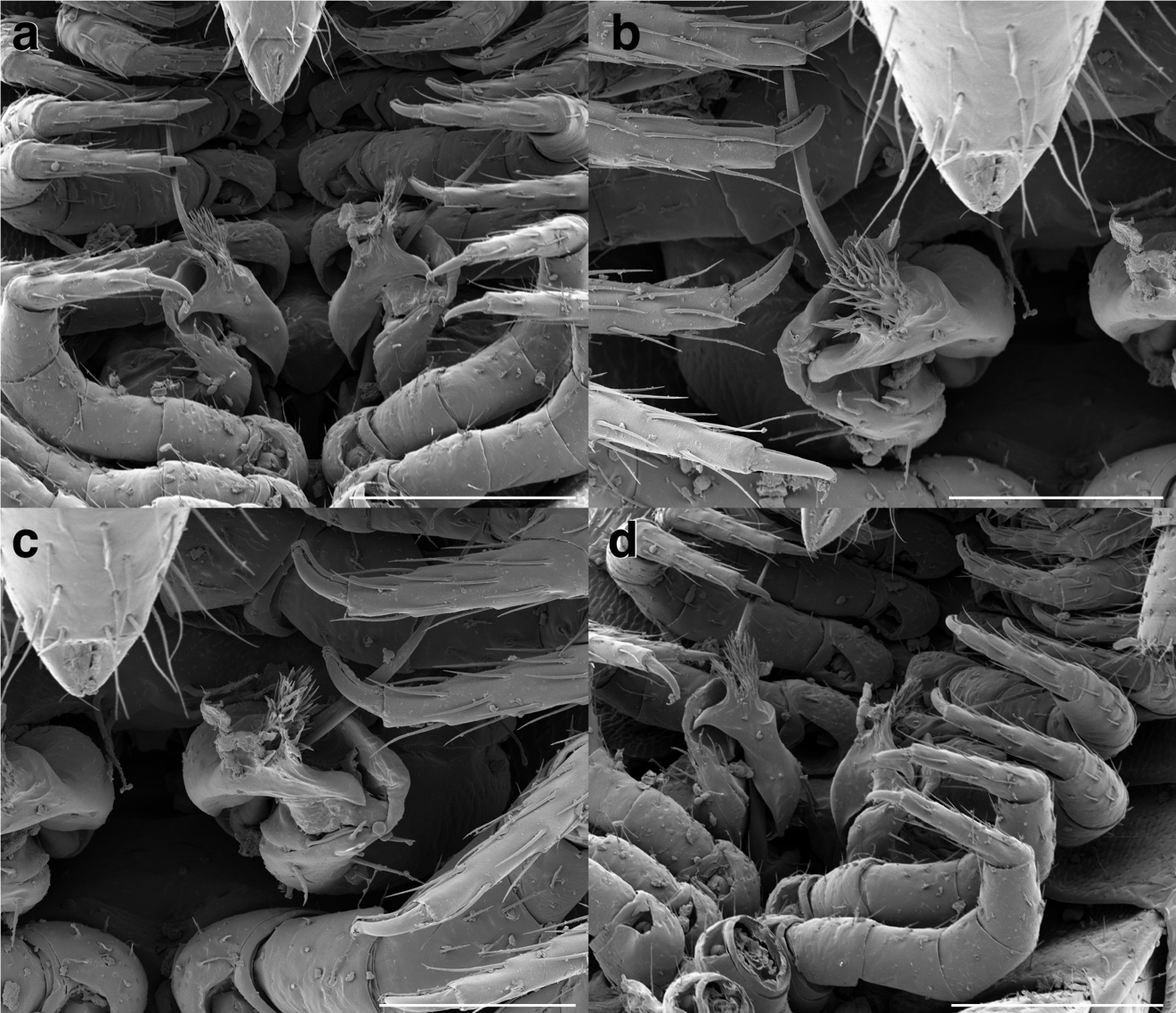


**Supplementary figure S4.** *Eumillipes persephone* male holotype (T147101). A. Ventral view of gonopods. (Scale bar = 200 μm). B. Ventral view of right gonopods. (Scale bar = 100 μm). C. Ventral view of left gonopods. (Scale bar = 100 μm). D. Oblique-ventral view of gonopods showing anterior gonopods distinctly bifurcated into two processes. (Scale bar = 200 μm).

**
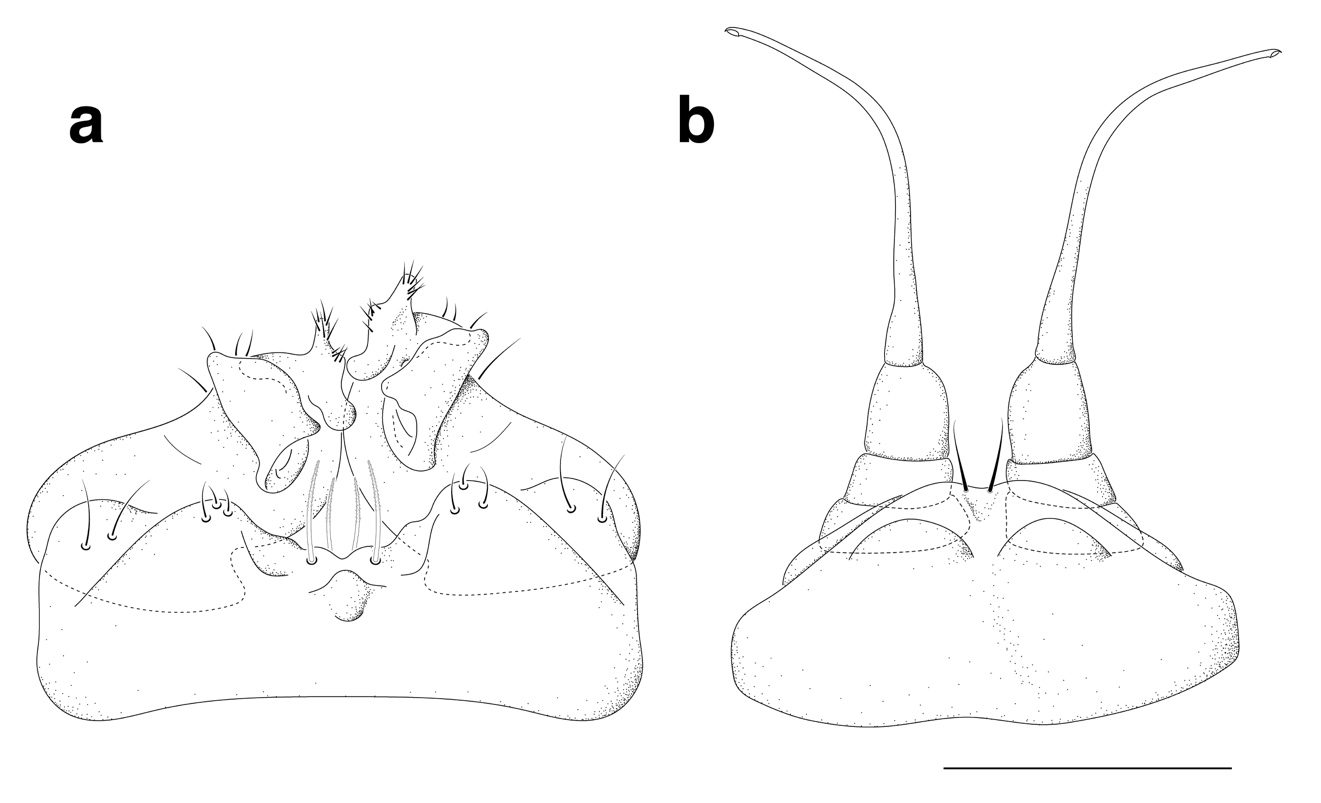
**

**Supplementary figure S5.** *Eumillipes persephone* male paratype (T147100). Anterior view of anterior and posterior gonopods. A. Anterior gonopods (9th leg pair). B. Posterior gonopods (10th leg pair). (Scale bar = 200 μm).


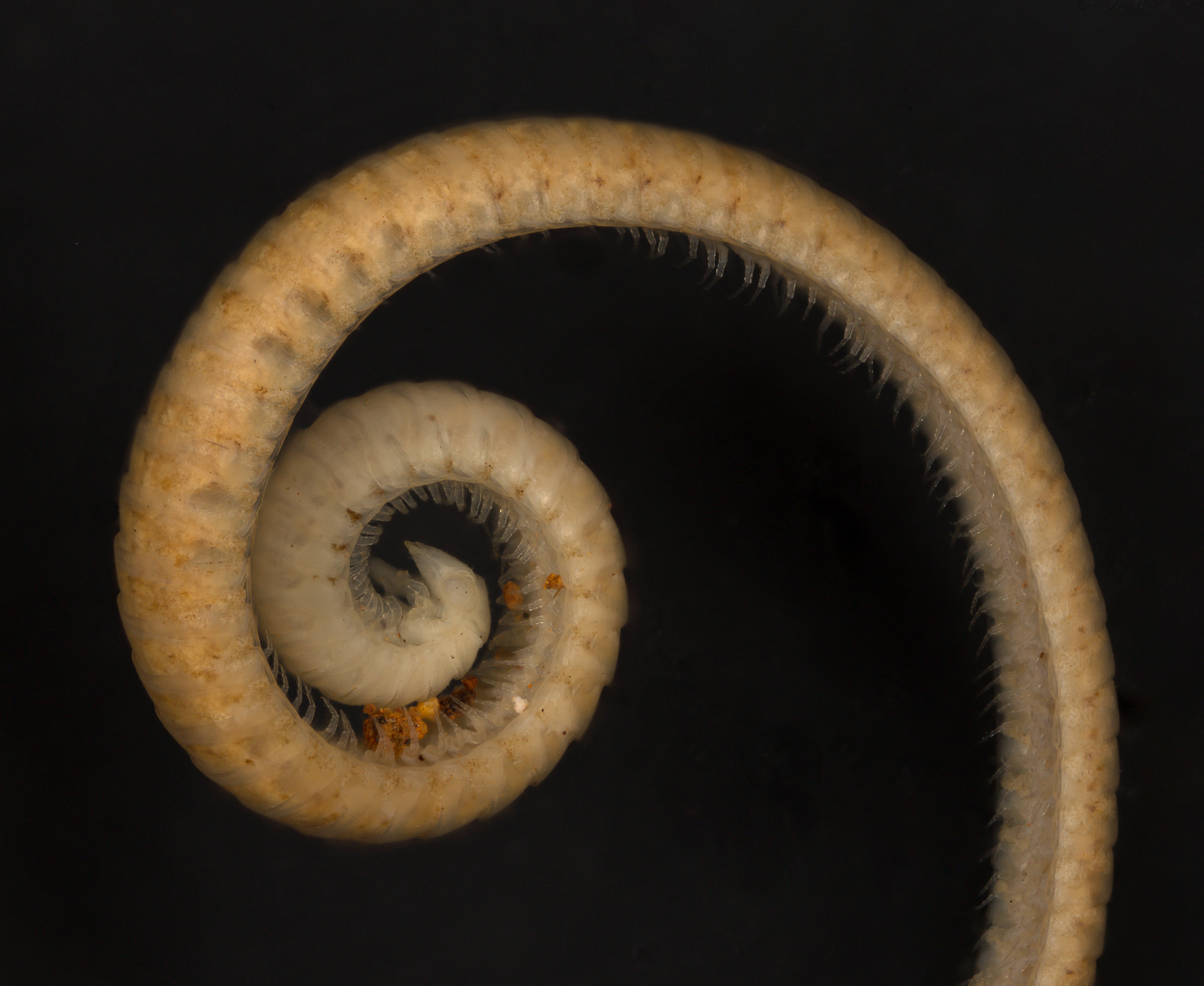


**Supplementary figure S6.** Anteriormost 64 segments of a female paratype of *Eumillipes persephone* with 330 rings and 1,306 legs (T147124).


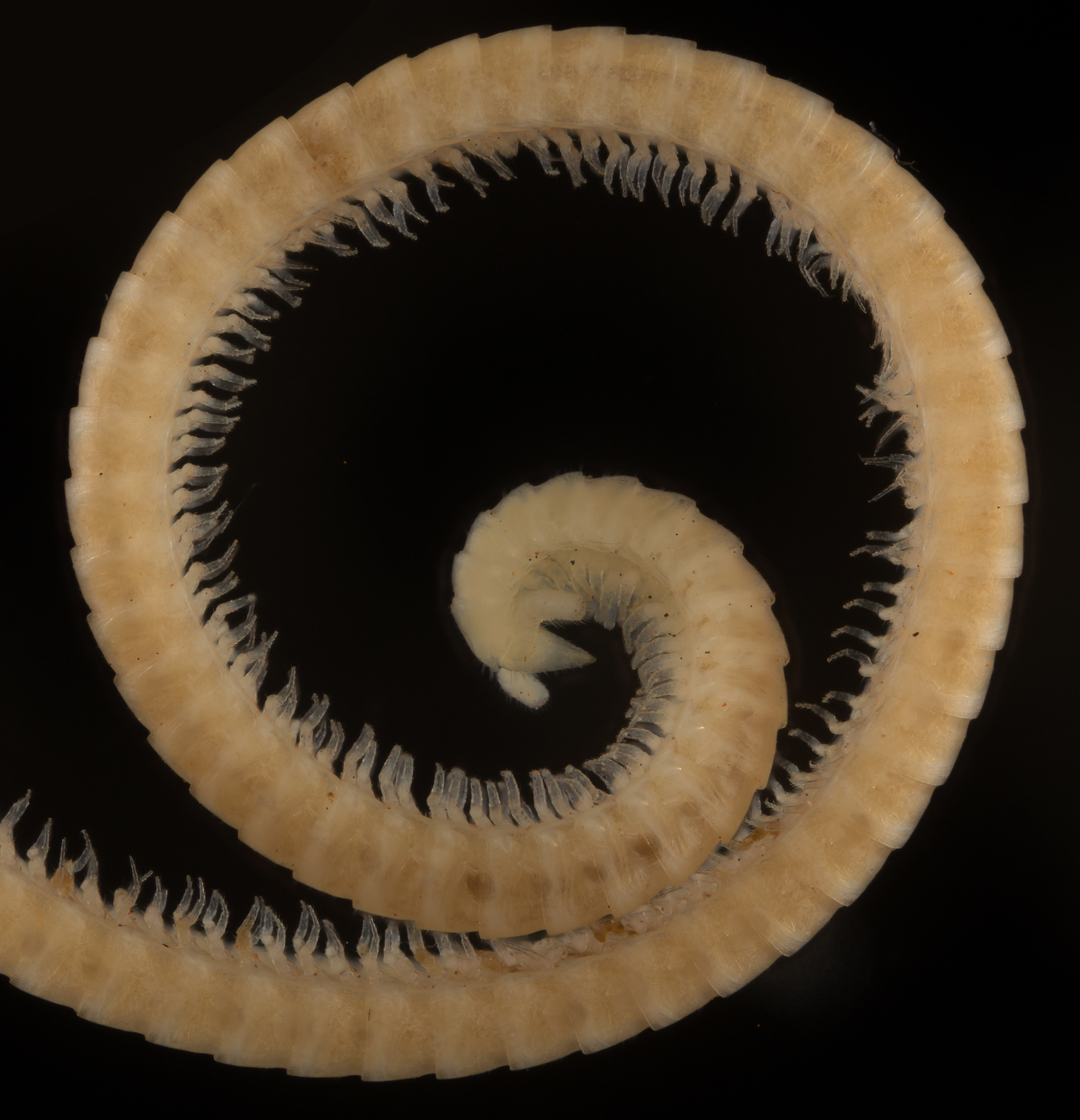


**Supplementary figure S7.** Anteriormost 66 segments of the male holotype of *Eumillipes persephone* with 198 rings and 778 legs (T147101).


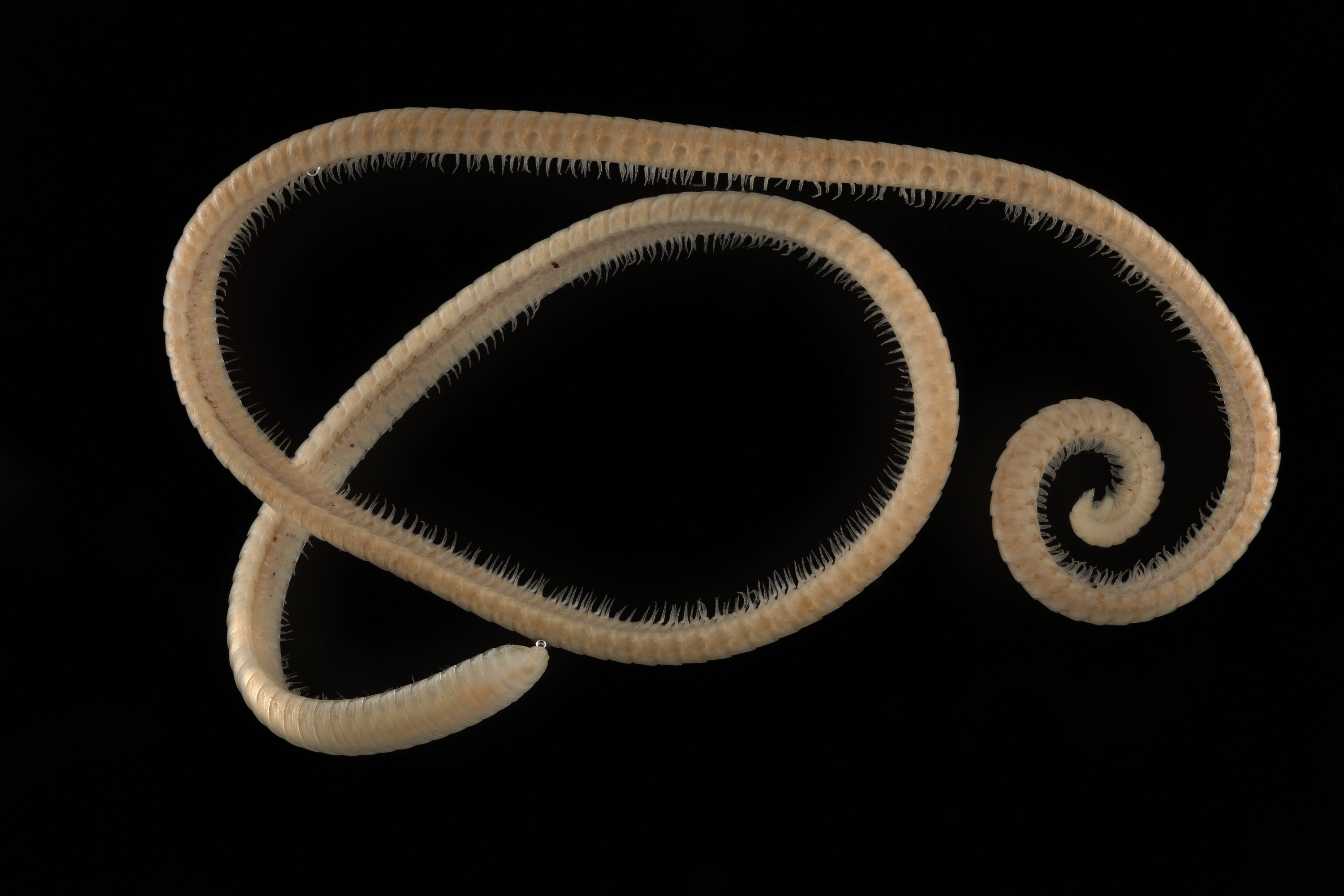


**Supplementary figure S8.** *Eumillipes persephone* male paratype specimen with 208 rings and 818 legs (T147100).


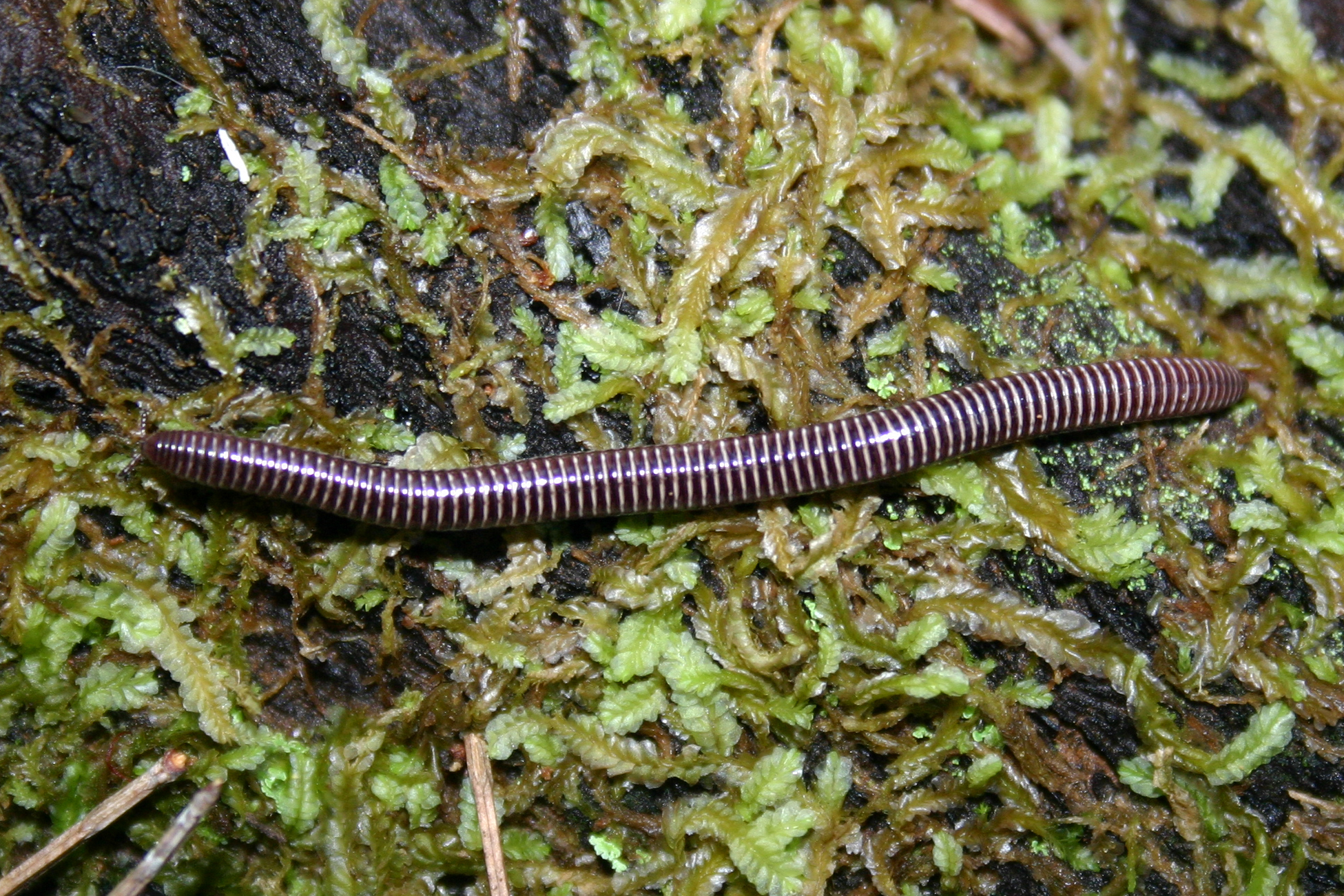


**Supplementary figure S9.** An undescribed genus and species of surface-dwelling polyzoniidan millipede from Walpole, Australia with 90 segments (family Siphonotidae).


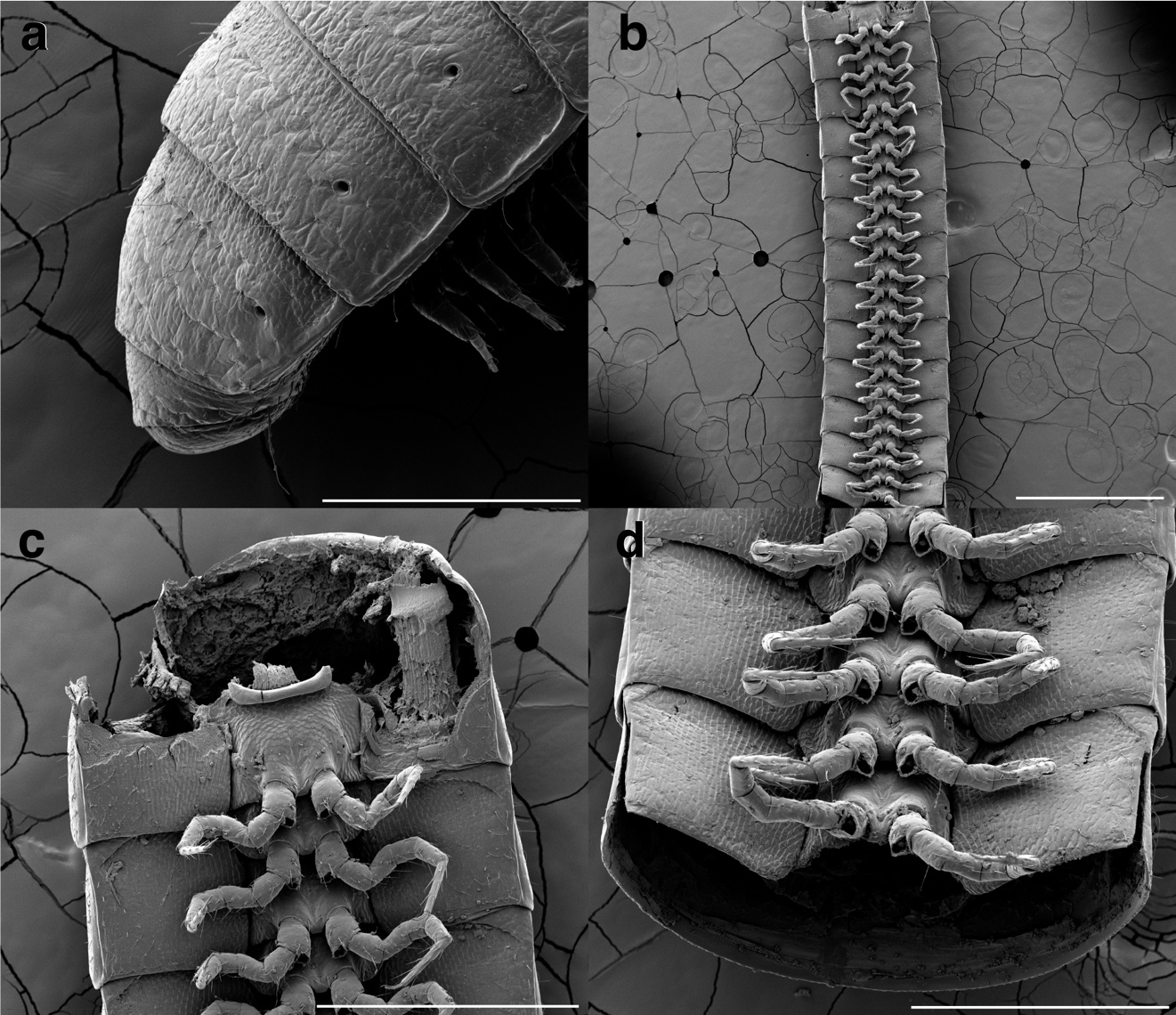


**Supplementary figure S10.** *Eumillipes persephone* male holotype (T147101). A. Posterior-most 4 segments + telson, lateral view, showing telson a complete ring around the anal valves. (Scale bar = 300 μm). B. Mid-length trunk segments, ventral view. (Scale bar = 1 mm). C. Mid-length trunk segments, ventral view, showing free sternites and pleurites (large longitudinal muscle visible through fractured pleurite). (Scale bar = 500 μm). D. Mid-length trunk segments, ventral view, showing free sternites and pleurites, and large coxal/eversible sacs. (Scale bar = 400 μm).

**
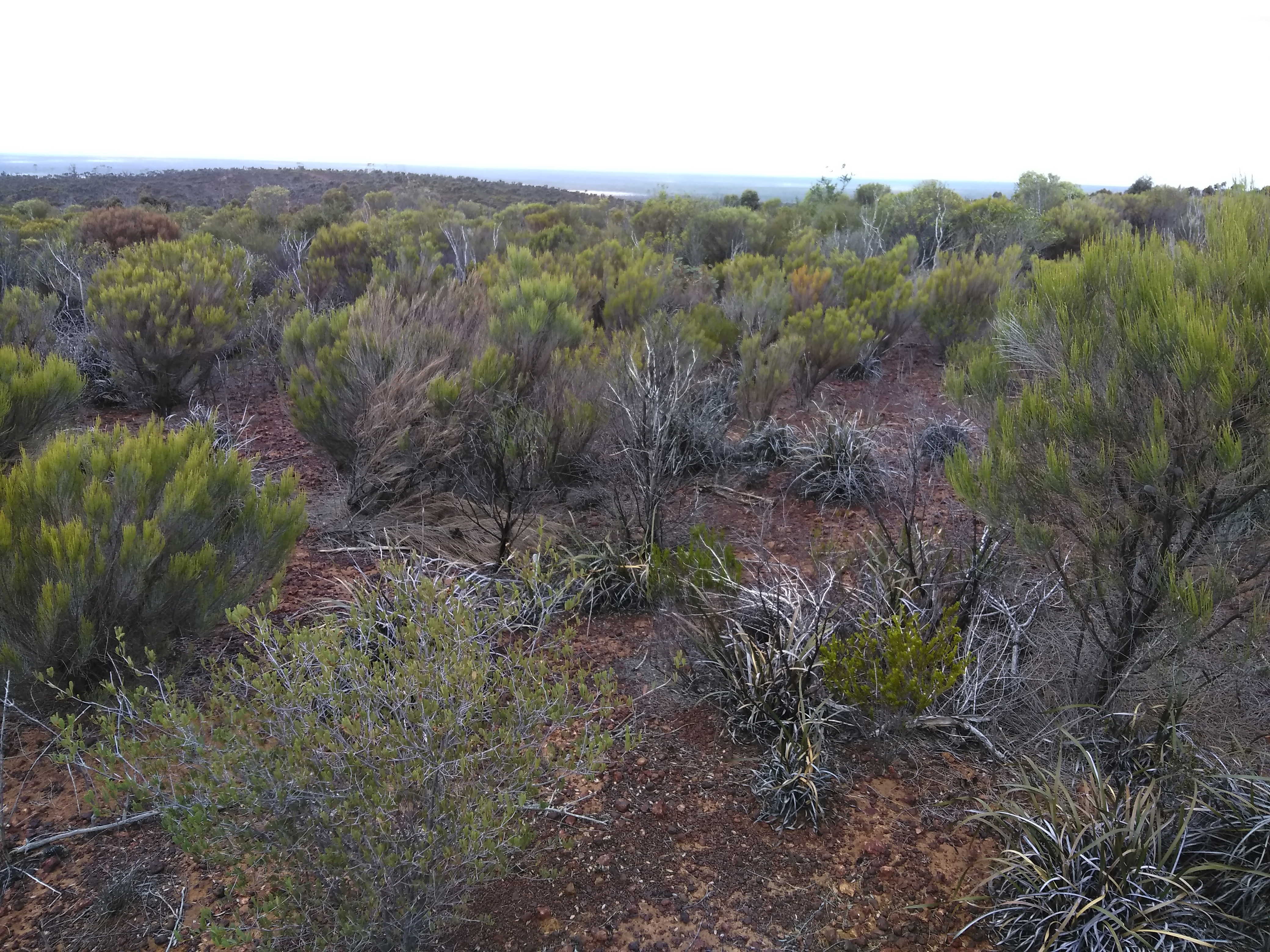
**

**Supplementary figure S11.** Sheoak shrubland on the surface of *Eumillipes persephone* habitat.

**Table S1.** Measurements of type specimens. Abbreviations: p = number of podous rings, a = number of apodous rings, l = number of legs; the following in millimeters — BL = body width, HW = head width, HL = head length (measured from the posterior margin of the antennal socket to the labral tip), AW = antenna width, CW = collum width, W1 = segment width, H1 = segment height.

| **Specimen** | **sex** | **p** | **a** | **l** | **BL** | **HW** | **HL** | **AW** | **CW** | **W1** | **H1** |
| --- | --- | --- | --- | --- | --- | --- | --- | --- | --- | --- | --- |
| T146684 | female | 252 | 1 | 998 | 72.9 | 0.21 | 0.35 | 0.13 | 0.57 | 1.00 | 0.54 |
| T147124 | female | 329 | 1 | 1306 | 95.7 | 0.24 | 0.44 | 0.12 | 0.53 | 0.95 | 0.51 |
| T147100 | male | 207 | 1 | 818 | 59.4 | 0.25 | 0.37 | 0.15 | 0.55 | 1.00 | 0.43 |
| T147101 | male | 197 | 1 | 778 | 54.7 | 0.21 | 0.40 | 0.12 | 0.52 | 0.92 | 0.43 |

**Supplementary References**

1. Rodriguez, J. *et al.* Step-wise evolution of complex chemical defenses in millipedes: a phylogenomic approach. Sci. Rep. 8, 1-10. https://doi.org/10.1038/s41598-018-19996-6 (2018).
2. Maddison, W. & Maddison, D. R. Mesquite: a molecular system for evolutionary analysis. Version 3.5. http://mesquiteproject.org/ (2010).
